# Changes in Indocyanine Green Lymphography Patterns after Physical Treatment in Secondary Upper Limb Lymphedema

**DOI:** 10.3390/jcm9020306

**Published:** 2020-01-22

**Authors:** María Elena Medina-Rodríguez, María de-la-Casa-Almeida, Jesús González Martín, María Hermida Anllo, Esther M. Medrano-Sánchez

**Affiliations:** 1 General Hospital of Gran Canaria Dr. Negrin, Barranco de la Ballena s/n, Las Palmas de Gran Canaria, 35010 Las Palmas, Spain; mariaelena.medina@ulpgc.es; 2 Department of Medical and Surgical Sciences, University of Las Palmas de Gran Canaria, Campus de San Cristóbal, 35016 Las Palmas, Spain; 3 Department of Physiotherapy, University of Seville, C/Avicena s/n, 41009 Seville, Spain; emedrano@us.es; 4 Research Unit of General Hospital of Gran Canaria Dr. Negrin. Barranco de la Ballena s/n, Las Palmas de Gran Canaria, 35010 Las Palmas, Spain; josu.estadistica@gmail.com; 5 Department of Vascular Surgery of General Hospital of Gran Canaria Dr. Negrin, Barranco de la Ballena s/n, Las Palmas de Gran Canaria, 35010 Las Palmas, Spain; maheran@live.com

**Keywords:** breast cancer lymphedema, indocyanine green, lymphography, manual lymphatic drainage

## Abstract

Indocyanine green (ICG) lymphography is used to evaluate the lymphatic function before and after pneumatic compression or post-manual lymphatic drainage. The aim of this study was to ascertain the changes in the fluoroscopic pattern produced by the provision of complex physical therapy. This prospective analytic (pretest-posttest) study was conducted in 19 patients with upper lymphedema secondary to breast cancer. Nine patients were excluded due to ICG found after 3 weeks. The ICG patterns were analyzed under basal conditions and after three weeks of treatment. After the treatment, 45% of the patients presented tracer remains in the affected limb, and this finding was significantly related to time of the lymphedema development. In one subject, the patterns remain unchanged or cannot be defined. Three of the ten patients observed present the worsening of at least 1 of the patterns and in the rest of the subjects, six cases, the improvement of the patterns is observed. In 60% of the cases, the most severe pattern reversed towards slight (splash) cases, and moderate cases reversed towards a slight case in 70% of cases. Therefore, after treatment with complex physical therapy, the pathological patterns observed in the pretest, which evolved positively, reverted their severity toward milder disease patterns or towards normality.

## 1. Introduction

Secondary lymphedema of the upper limb related to cancer treatment is characterized by the accumulation of protein-rich fluid in the interstitial space due, to the decreased transport capacity of the lymphatic system, after dissection of the axillary lymph nodes and radiation to the lymph nodes [1,2,3].

In the earliest lymphedemas, although the circumference has not increased significantly [4], symptoms may appear, and they are interpreted as the sub-clinical manifestation of lymphatic system disorder [5]. This initial dysfunction can be detected through complementary tests such as indirect lymphography [6] or indocyanine green (ICG) lymphography [7]. In these tests, the presence and extension of dermal reflux is interpreted as a diagnostic criterion of lymphedema [7,8,9,10]. This reflux is a black flow of lymphatic fluid towards the superficial lymphatics, remaining in the epiphasic space of the skin and subcutaneous tissue, as a result of lymphatic system dysfunction [11,12,13]. 

Under normal conditions, ICG lymphography shows a linear pattern of superficial lymphatic channels that are straight and well-defined [7,11,14]. In diseased situations, the image of the dermic reflux progresses, as does the severity of the system involvement, towards a splash pattern and over time toward a stardust and diffuse pattern [13,15,16]. One single patient may show several dermal reflux patterns [17].

The International Society of Lymphology recommends that lymphedema be treated by combined physical therapy [18] or Complex Decongestive Physical Therapy (CDT), which is considered the standard lymphedema treatment [19]. Treatment should start in the early phase [7,20], as in chronic cases, physical therapy is unlikely to completely reduce the lymphedema [21] and surgery might be required. The best time to start physical therapy treatment of edema should be when ICG lymphography detects splash patterns [22].

CDT involves two stages: the intensive phase, which pursues the largest reduction in volume and the maintenance phase, which aims to preserve and optimize the results obtained in the intensive phase [18].

The first phase includes skin care, lymphatic drainage massage, multilayer compression bandages and exercise [19,23]. Pressotherapy is sometimes used along with CDT. In the second phase, a short elastic sleeve replaces the compression bandage, and lymphatic drainage is conducted when necessary [18].

ICG lymphography has been used to determine the most appropriate therapeutic strategy, whether surgical or conservative [4,24,25], and to monitor treatment response in patients with lymphedema [14,26,27]. Published studies have reported this technique to be useful when assessing the lymphatic system’s contractile function, before and after pneumatic compression therapy [26] or after the Manual Lymphatic Drainage (MLD) technique [28,29,30]. Using ICG lymphography, some authors even confirm how a single session of MLD improved the speed of lymphatic flow and decreased propulsion time [27].

In our study, we aimed to determine whether an ICG tracer completely disappears after three weeks of intensive physical treatment and if the results is influenced by factors such as age, body mass index and the time of the evolution of the lymphedema.

We wanted to determine if a three-week intensive physiotherapy treatment produces changes in fluoroscopic patterns in the upper extremities with secondary lymphedema after axillary lymph node resection. 

## 2. Methods 

### 2.1. Study Cohort

This prospective analytic (pretest-posttest) study was conducted at the Dr. Negrín University Hospital of Gran Canaria (H.U.G.C.Dr. Negrín), Las Palmas, Spain, from January to July 2017. We evaluated the 19 upper limbs from 19 women diagnosed with secondary upper limb lymphedema after axillary lymph node resection for breast cancer. The diagnosis was obtained from the medical history and physical examination (perimetric and volumetric differences between the limbs). The patients were on the waiting list for physical treatment at the Lymphatic Pathology Unit of the Rehabilitation Service of H.U.G.C. Dr. Negrín. The patients were included in the study according to their order of registration on our waiting list, and after verifying that they met the selection criteria. Ten of them referred have received physical treatment at some point in their lives. The Committee of Ethics in Biomedical Research (CEIB) of the H.U.G.C. Dr. Negrín approved this study protocol (Code CEIC Negrín 170022), as well as the Spanish Agency of Medicines, which classified it as non-observational without medication.

All study participants freely provided their informed consent prior to participation. The patients excluded from the study (1) were under 18 years of age, (2) had secondary edema due to another cause other than the treatment of breast cancer, (3) had, or were suspected of having, deep vein thrombosis, (4) presented an allergy to iodine or any of its derivatives, (5) had not finished or (6) had not signed the informed consent before the beginning of the study.

The average age of the patients was 59 years (53–68 years), with 8 of the 19 volunteers presenting stage II A and 11 presenting stage II B lymphedema according to the classification proposed by the International Society of Lymphology (ISL) [31]. Respect to the time elapsed from surgery to the appearance of edema, 8 subjects developed their lymphedema during the first year after surgery, 2 after two years, 3 at three, one after 5 and 4 after more than ten years (10, 11, 12 and 14 years later).

The percentage of severity was calculated by the formula recommended by Ferrandez et al. [28]: Severity = (volume of the affected side − volume of the healthy side)/volume of the healthy side. The severity percentage is obtained by multiplying this result by 100. Depending on the percentage of the volume difference between the members, the level of severity of lymphedema is classified as: normal (0–10%), mild (10–20%), moderate (21–40%) and severe (>40%) [8,31]. The level of severity of the lymphedema at the beginning of the study was mild in 31.6% of the subjects (*n* = 6), moderate in 37% (*n* = 7) and most severe in 31.6% (*n* = 6). 

### 2.2. Intervention 

The study was structured in three phases: pre-treatment evaluation, three-week intensive physiotherapy treatment and post-treatment evaluation.

#### 2.2.1. Phase 1: Pre-Treatment Assessment

First, the physiotherapist tabulated 11 reference points for measuring the perimeter of each limb. In the hand, according to Villaverde’s recommendation [5], the marking point behind the metacarpophalangeal joints was registered. The wrist joint was marked by a line between the radial and ulnar styloid processes. At the elbow, the junction line between the epicondyle and the epitrochlea was marked. To mark the forearm reference points, the ulnar styloid and epicondyle were used as the starting point [5]. From these references, marks were made every 4 cm [5,12], recording four measuring points on the forearm (situated to 4, 8, 12 and 16 cm. over the wrist) and four others on the rest of the arm, covering the regions located from the elbow to the armpit (to 4, 8, 12 and 16 cm over the elbow).

Each reference was identified on the anterior and posterior side of the extremity with white adhesive tape, opaque to infrared light. Once the references were marked, the physiotherapist measured each point with a tail strap while the patient was in the supine decubitus position. Given the high intra-observer’s reliability of this measurement system [5,7,32], the patient was evaluated by a single physiotherapist. Three measurements were recorded at each of the points and the value of the mean was recorded. 

Each point was measured, three times and the average of the three measurements was obtained by a physiotherapist. Subsequently, the volumes (V) of the extremities were calculated using the formula V = P^2^/π [5], where P is the segment’s perimeter measured every 4 cm. The percentage of lymphedema severity was calculated as recommended by Ferrandez [28].

A vascular surgery specialist proceeded to inject 0.3 mL of 25 mg ICG solution (Verdye^®^, Waas Anita, S.A. Diagnostic Green GmbH, Aschheim-Dornach, Germany) in 5 mL of 5% glucosylated water in the second and fourth interdigital spaces in the hand of the affected member. The procedure was conducted with the patient lying in the supine position and with the limb resting on the stretcher.

After injection, patients were asked to remain motionless for five minutes and then performed isolated movements of flexion and extension of the fingers of the affected limb for another five minutes.

At 90 min, post-ICG injection, under basal conditions, a team formed by a vascular surgeon and a physiotherapist observed the presence of the ICG tracer through an infrared camera (Photodynamic Eye, Hamamatsu Photonics K.K., Hamamatsu, Japan). A third observer, a physiotherapist, verified the correct visualization and recording of the images obtained. 

The images were stored for further analysis. The team checked the type of fluoroscopic pattern present in the same areas that were selected for the perimeter measurement. The patterns analyzed in the anterior and posterior faces of the 11 reference regions of the affected extremity, were defined as linear, splash, stardust, and diffuse [13]. When the tracer was not visualized, the pattern was recorded as none.

To prevent contamination of the patient’s skin with ICG when injecting the tracer, during observation or by the patients themselves, all those involved (participants, medical personnel and physical therapist) wore latex gloves and avoided contact with the injection area.

#### 2.2.2. Phase 2: Intensive Physical Therapy Treatment

This treatment was provided by two specially trained physical therapists from our rehabilitation unit. All the patients received 15 sessions involving skin care, multi-layer compression bandages, pressotherapy, MLD and exercises. The sessions were scheduled on a daily basis over three consecutive weeks. 

The drainage treatment lasted 60 min and comprised the neck, armpits, chest, back, and Mascagni and Caplan pathways [33]. These regions and the areas of the extremity free of edema were only stimulated by means of call maneuvers, according to the instructions of the Leduc Method^®^. The lymphedematous regions of the extremity were approached with resorption maneuvers [34].

After the MLD, a pressure therapy pump was applied to the affected member for 30 min with a pressure of 40 mmHg [19]. The skin was then hydrated, and a multilayered bandage was applied to the affected member with short elastic bandages. Active mobilization exercises were performed on all limb joints for 10 min. 

The patients wore the bandage day and night, throughout the treatment period and performed global mobilization exercises of all the joints of the limb, for 10 min, twice per day at home. 

In the following session, immediately after this phase, a post-test assessment was completed.

#### 2.2.3. Phase 3: Post-Test Evaluation

In this second assessment, in basal conditions, the circumference was measured on all the participants. 

An infrared camera (Photodynamic Eye, Hamamatsu Photonics K.K., Hamamatsu, Japan) also verified ICG tracer presence in the extremity. 

In those patients in whom the tracer had been completely reabsorbed by the lymphatic system, a second fluoroscopic evaluation was completed, following the same exploratory procedure described in the first phase.

To check the evolution of the ICG lymphography pattern after treatment, we analyzed the criterion change of the initial-final pattern, after physical therapy treatment, and established 4 possible outcomes: improvement, worsening, no variation and no evaluation. 

The “improvement” result indicated a change from the initial to a milder pattern, after physical therapy treatment, whereas the “worsening” result was interpreted as lymphedema aggravation, according to Yamamoto’s classification [13].

The “did not vary” result indicated that the initial ICG pattern remained unchanged after physical therapy treatment.

If the tracer was not detected in an area, it was identified as a mute zone, and was called a non-identifiable pattern or none (0). In the presence of a mute zone, in the pre or post-test, the result was considered as not evaluated.

To calculate the frequencies and percentages in the post-treatment pattern changes, the cases in which the result of the treatment was not assessed were excluded due to the presence of a mute zone or unidentifiable pattern in the pre- or post-test.

We analyzed the finding of tracer remains, after three weeks of intensive physical therapy treatment related to the variables: age, body mass index (BMI), time of the evolution and level of initial severity of the lymphedema 

### 2.3. Statistical Analysis

The mean and 25th and 75th percentiles were calculated to describe the quantitative variables. We calculated the frequency and the percentage in the qualitative variables and the Mann-Whitney U non-parametric test was used, due to the small size of the groups, to compare the influence of numerical variables such as age or body mass index between groups G1 (No Tracer Remains After Treatment; *N* = 10) and G2 ((Present Tracer Remains After Treatment; *N* = 9). 

The exact Fisher´s test was used to compare qualitative variables, and the chi-squared test for trend was used to compare ordinal variables, such as the level of initial severity of lymphedema (mild, moderate and severe) and dichotomous variables (visible and no visible groups) *p*-values < 0.05 were considered significant. The statistical program used was Core Team (2018) [35].

## 3. Results 

**Figure 1:**
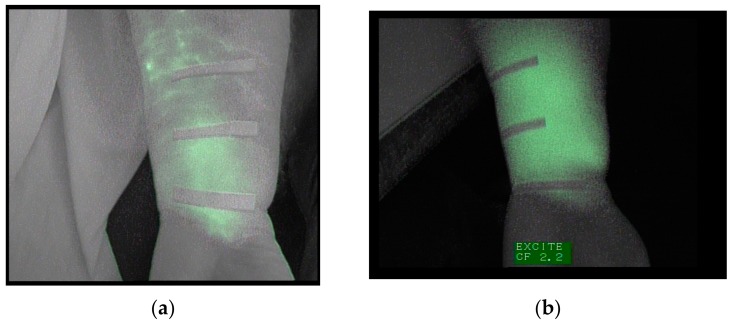
(**a**) Image of the forearm captured under basal conditions. (**b**) Image of the same forearm, taken after the treatment, depicting a widespread diffuse pattern.

Regarding the first objective of this study to check if after three weeks of intensive physical treatment the ICG tracer completely disappeared of the 19 patients studied in the pretest, 9 (47%) showed trace remains (ICG) in the extremity. One of these cases is shown in Figure 1. At the first observation under basal conditions, the forearm of this patient showed a diffuse pattern in the palmar region of the wrist and 4 cm away. At 8 cm from the wrist, a stardust pattern was observed (Figure 1a).

After three weeks of treatment, this subject presented a diffuse pattern along the forearm, and the stardust pattern was replaced by a diffuse pattern (Figure 1b). This patient was excluded from the second fluoroscopic exploration. 

In line with the first objective, the relationship between the indocyanine remaining after three weeks of treatment, and age, BMI, and were not significant (*p* = 0.7, 0.71 respectively).

The t factors that showed a significant relationship with the presence of tracer after three weeks of intensive physical treatment, were the level of initial severity (*p* = 0.021) and the time of the evolution of the lymphedema (<0.001). For the analysis of this relationship, a linear trend was used. The results of the analysis of this relationship are shown in Table 1 and Table 2. 

Eight of the nine subjects in group 2 had a clinical diagnosis of lymphedema for more than 1 year and received physical therapy at some point in their lives. The 2 subjects in group 1, included in the study, who had more than 1 year of clinical diagnosis of lymphedema were not previously treated with physiotherapy.

We explored 11 anterior and posterior regions of the extremity of each of the 10 patients, for a total of 220 regions to determine if a three-week intensive physiotherapy treatment induces changes in fluoroscopic patterns in the upper extremities with lymphedema secondary for the treatment of breast cancer after axillary lymph node resection.

All data of the changes observed in the anterior and posterior regions of each studied subject can be found in Tables S1–S4 of the supplementary material.

In one subject, the patterns remain unchanged or cannot be defined. Three of the ten patients observed present the worsening of at least 1 of the patterns and in the rest of the subjects, six cases, the improvement of the patterns is observed.

The change of the initial ICG lymphography pattern after treatment, in all the body regions explored, was improvement in 26%, worsening in 7%, no variation in 32%, and in no evaluation in 35%.

The most change was observed in the elbow region. The more distal regions, with respect to the elbow, evolved more favorably. 

**Figure 2:**
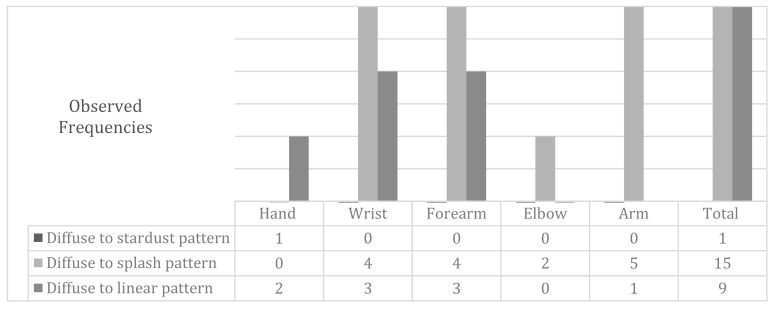
Modifications in the diffuse pattern following the treatment of physiotherapy by body regions.

In order to unify the observed data and facilitate its understanding, we decided to show in the following results tables (Figures 2, 4 and 6) the changes in the patterns by wider regions, but with the inclusion of the tables in this document, as Supplementary Material, the reader interested can check the changes in all regions.

For areas whose patterns remained unchanged, after three weeks of intensive treatment, 60% showed a linear pattern (normal), 31% mild, 13% stardust, and 12% diffuse.

The analysis of the initial patterns that improved after three weeks of treatment showed that the following:

The diffuse (severe) pattern evolved to a splash pattern (mild) in 60% of cases, and 36% evolved to a linear pattern. Only one case (4%) evolved to a moderate or stardust. 

Then, in Figures 2, 4 and 6 it can be analyzed, in the different body regions, and in terms of absolute frequencies, the number of times the change in the indicated pattern could be observed.

The behavior of the improvement in the diffuse ICG pattern by body regions after physiotherapy treatment depicted in Figure 2. In one case, the hand evolved toward a stardust pattern.

**Figure 3:**
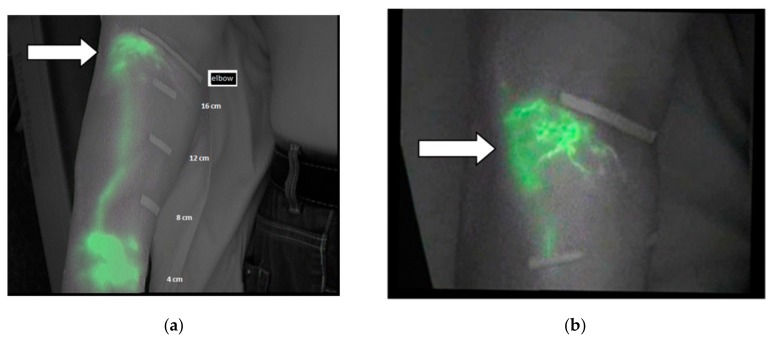
(**a**) Diffuse pattern under the elbow before the treatment and (**b**) splash pattern after the treatment as indicated by white arrows.

In Figure 3a, a diffuse pattern is observed in the region of the forearm located under the elbow. Figure 3b shows the same area with a splash pattern after the treatment. 

**Figure 4:**
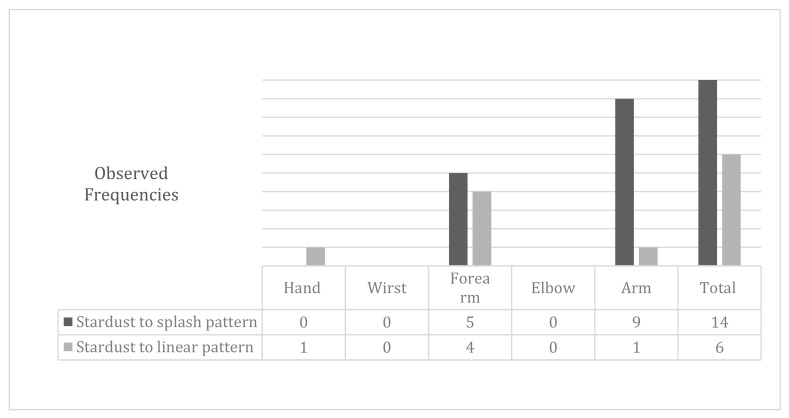
Modifications to the stardust pattern after physiotherapy treatment.

The stardust pattern (moderate) evolved in 70% of cases, toward a slight pattern (a splash pattern) and the rest evolved toward a linear pattern. The initial stardust pattern, showed before the intensive treatment, improved to one splash pattern, as shown by the data in Figure 4. The changes in the stardust pattern along the affected limb are also shown in Figure 4.

**Figure 5:**
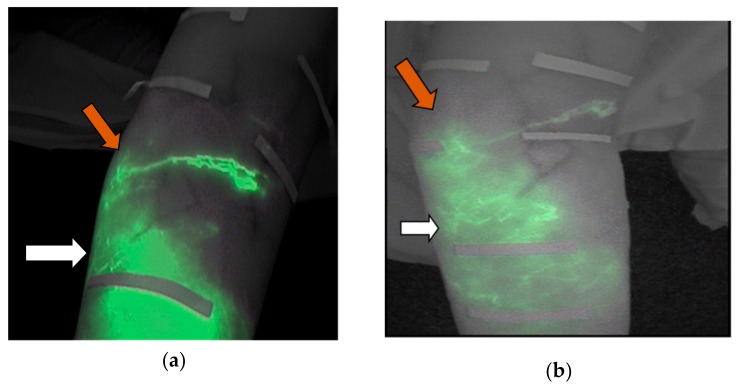
(**a**) Image captured before the treatment. The white arrow shown indicates the stardust pattern and the orange indicates a splash pattern. (**b**) Image captured after the treatment. The white arrow indicates a splash pattern and the orange arrow shows a linear pattern.

Figure 5a,b depicts the evolution in the same case of two forearms’ patterns a splash pattern to a linear pattern (white arrow) and a stardust pattern to a splash pattern (orange arrow).

**Figure 6:**
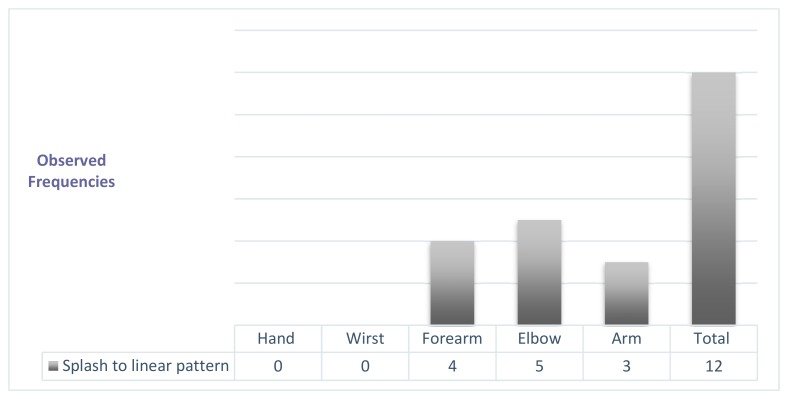
Modifications of the splash pattern after physiotherapy treatment.

The splash pattern evolved preferably towards normality in the most distal body regions (Figure 6).

As can be seen in Table S4 of the supplementary material, of Results in absolute frequencies of the changes of ICG pattern in the body regions assessed, after the Physiotherapy treatment, the changes in the regions situated from the hand to the elbow, usually become more frequently linear, normal patterns. On the other hand, the patterns of the regions above the elbow tend to improve to milder patterns.

## 4. Discussion

In this study, we observed the remains of the green tracer of indocyanine after three weeks of intensive treatment in 47% of the patients evaluated through ICG and identified, as is expected, a relationship of this event with the time of the evolution of the lymphedema. We emphasize how the severity of pathological patterns, which evolved positively after intensive physiotherapy treatment, reversed in 26% of the observed patients mainly toward mild or normal patterns.

Given its safety level, ICG lymphography has been recommended as a method for continuous and repeated examination of lymphedema [36] for the evaluation of its response to treatment [6]. We have not found references to a recommended time between observations. Narushima et al. [7] reported that the places where the tracer is injected are dyed green, and that it disappears after two weeks, although the camera could detect it for more than a month after the injection.

We have not found any published data on tracer remains, after intensive treatment of physiotherapy, as was observed in 47% of our study population. Based on this finding, we recommend that future research using this technique as a system of control response to treatment consider our finding in the planning of successive explorations.

We analyzed the relationship of age, BMI, initial severity, and time of the evolution of the lymphedema in the visualization of remaining ICG tracer in the lymphedematous extremity with the emergence of lymphedema [37] and the response to physical therapy has been reported [21,38,39]. The variables significantly associated with tracer presence were the level of initial and evolution time of the lymphedema. With high probability (80%), after three weeks of intensive treatment, in lymphedemas lasting one year or older, the tracer continued to be visible. Liao et al. [40] found that the oldest lymphedemas responded poorly to complex physical therapy, so we postulate that the incomplete resorption of the ICG tracer, after three weeks of intensive treatment, could be a sign of the inability of the system to reabsorb the high molecular weight substances in the interstitial space, despite physical therapy.

In the lymphedemas of longer duration, more than one year, three weeks of intensive physical treatment were not sufficient to achieve the reabsorption of the ICG tracer. It is necessary to investigate whether these lymphedemas need longer physical treatment times or that their response to the physical treatment is not as effective. We hypothesize that the second answer is the correct response to this question. Future observations should investigate the average time for the tracer to completely disappear in the oldest lymphedema both after and without physical treatment.

The second finding of our study is the changes induced by a three-week intensive treatment of physiotherapy on the initial ICG patterns in the evaluated patients in whom no trace remains were found. A total of 10 patients were diagnosed with upper-limb secondary lymphedema after breast cancer treatment, coinciding with the findings reported by Akita et al. [17]. Different initial ICG lymphography patterns were found in the same affected extremity, and we verified that after intensive physiotherapy treatment, 26% of the initial pathological patterns improved, 7% worsened, and 32% remained unchanged.

The regions whose initial patterns were not affected after intensive treatment often presented a normal initial pattern.

After treatment, some regions evolved positively: 47% toward a linear pattern (normal), 51% toward a slight pattern, and only one case with a severe or diffuse initial pattern evolved toward a stardust pattern. Despite expectations that the patterns evolved toward the immediately inferior pattern in the severity scale, in our study, the improvement tended to be toward mild patterns or even normality.

In line with our finding, we found references to the evolution of pathological ICG patterns in the published literature [7,17] similar to those of the splash or stardust patterns, and to the recommended therapeutic strategies according to the type of ICG pattern [17].

Mihara et al. [36] stated that the splash pattern should be treated, since they found that it reverts to a linear pattern after treatment. This recommendation is shared by other authors [18,38] who see the need to approach the splash pattern with physical therapy.

Akita et al. [17] stated that physical therapy for stardust pattern with moderate severity, based on manual lymphatic drainage, skin care, and elastic stockings, was not able to convert this pattern to a slight, splash, or normal pattern. Narushima et al. [7], in their study on lymphedemas of lower limbs secondary to gynecological cancer, reported a trend toward the irreversibility of the stardust pattern if no proper treatment was provided, so only lymphedema anastomosis surgery would be the appropriate to reverse this pattern. Akita et al. [17] agreed that surgery is a viable treatment option in cases involving moderate-severity stardust patterns.

In our study, the initial severe or diffuse pattern, after three weeks of intensive physiotherapy treatment, improved toward a slight pattern (splash) in 60% of cases, toward a stardust pattern in 4%, and toward a linear pattern in 36% of cases. We emphasize the low proportion of cases with diffuse patterns that returned after physical therapy treatment toward a stardust pattern, which is the immediately lower stage in the lymphedema severity scale according to the classification proposed for the ICG lymphography [13]. The regions that presented an initial ICG pattern of moderate severity (stardust) and evolved positively did so in 70% of cases toward a slight pattern (splash), and the rest evolved toward normal.

Based on our results, we observed, in a case, that intensive treatment through complex physical therapy achieves the positive evolution of stardust pattern toward splash in secondary lymphedema involving breast cancer. This seems to challenge the results published by Akita et al. [17], which demonstrate the inability of conservative treatment to reverse this pattern in lower limb lymphedemas secondary to gynecological cancer. The findings of our study show that severe and moderate lymphedema with diffuse and stardust patterns after a three-week intensive physiotherapy treatment respond favorably and are transformed into mild splash patterns.

Although the splash pattern expresses early lymphatic dysfunction and tends to reverse naturally [17], in our study, it remained unchanged in 43% of cases after intensive therapy. The reversibility toward normality occurred in only 22% of the cases. Given our results, three weeks of intensive treatment may have been insufficient to reverse this mild pathological tendency toward normal. We have not found an explanation for this phenomenon in the literature consulted. We have simply shared our finding. After consulting the data tables and checking the severity of the patterns of the regions immediately superior to the observed areas, we did not find the presence of serious patterns that would hinder the evacuation of the tracer nor that these areas would occur more frequently in body regions determined. In response to this finding, we can only hypothesize that the persistence of the splash pattern, which demonstrates lymphatic disorder even in cases where clinical signs, would explain the persistence of the alteration of lymphatic dysfunction in those areas and the difficulty of restoring normal circulation.

The worsening of the areas in the forearm of 3 of the 10 subjects studied evolved into a fuzzy powder pattern. We observed the negative evolution of the forearm of one patient in five of the eight regions observed. We analyzed the characteristics of these patients in which the pattern worsened, including age, BMI, time of the duration, and severity percentage of the lymphedema. Of these, the only noteworthy aspect was BMI, which was higher (32.5; 32.9; 31.2) in cases where negative evolution was observed. Possibly, as Vignes et al. stated [41], the influence of increased BMI and excess fat accumulation in subcutaneous tissue negatively influenced the response to treatment in terms of reduction of the severity’s percentage of lymphedema. The same could occur regarding the negative evolution of the initial pattern in these cases.

In short, when the diffuse pattern after three weeks of physical treatment improved, it was not, as could be expected, toward the stardust pattern, which is immediately inferior in the severity scale, but toward a slight, splash pattern. The stardust pattern evolved with intensive treatment, as predicted, mainly toward the immediately inferior splash pattern on the severity scale. The splash pattern, despite treatment by intensive physical therapy for three weeks, did not evolve toward normality, but tended to remain unchanged.

Between the limitations, our study highlights the unavailability of the pre-operative baseline measures to collect the perimetric and volumetric differences between the members and calculate the severity of the lymphedema after the surgery. We did not visualize identifiable patterns in the pre-treatment in the most proximal regions of the extremities (located at 12 and 16 cm over the elbow), which prevented analyzing the effects of intensive therapy in these areas after the test. We do not know how to explain the presence of these mute areas in which the plotter was not visualized. Its presence in the hand makes us discard the idea that the period of time elapsed from the injection of the tracer until the observation has been insufficient and the cause of this phenomenon. 

The visualization of ICG tracer remains in 9 of the 19 patients studied was an unexpected and relevant finding, although this fact reduced the population in which the pattern changes were studied and could influence our study results. In future studies, a larger sample and a control group should be included, as recruiting at the time the study was impossible due to technical difficulties. Future studies should examine the time at which the tracer of the interstice disappears considering the variables described to systematize the methodology of routine exploration through ICG lymphography in these patients.

In our study, ICG lymphography allowed us to verify how the initial patterns responded to complex physical therapy. We suggest further studies that further test our hypothesis. The type of initial pattern could predict the lymphedema’s response to intensive physical treatment. Based on our study results, physical therapy demonstrates its efficacy in improving lymphatic lymphedema, this time through the ICG lymphography qualitative valuation. The improvement was assessed based on the ability of physical treatment to modify and improve initially pathological ICG patterns. New lines of research could analyze how different components of the tfc can modify the ICG patterns in lymphedema.

After analyzing our study results, we agree with other authors that ICG lymphography is useful for qualitatively assessing lymphedema and evaluating its response to physical therapy. Early intervention is stressed in treating lymphedema. Deciding on the most appropriate strategy, however, should be based on objective data such as the clinical lymphedema situation. ICG lymphography offers complementary information that facilitates decision making to better manage lymphedema. Its advantages over other methods of radiological exploration include its low cost [14], its safety [42], the possibility of studying the extremity in all directions [36], and no radiation exposure [36]. Thus, it provides a minimally invasive assessment [6] of the function and real-time lymphatic system architecture [20].

Society and health insurance managers expect the most effective interventions from health systems, and this is why we used physical therapy. The most suitable therapeutic options should be provided depending on the patient’s clinical situation and decisions should be made by objectively assessing the efficacy of all available therapeutic strategies.

Physical therapy has proven effective in daily clinical consultation mainly by measuring and comparing the perimetric and volumetric differences between the extremities. The ICG lymphography offers the possibility of knowing, in consultation, the real-time functional situation of the system to reabsorb the tracer, the evolution of ICG patterns after treatment and the response to treatment. Based on the results in the patients studied, we can expect a worse response to the physical treatment of the most evolved edema in terms of severity and time of evolution.

## 5. Conclusions

Visualization via ICG lymphography demonstrated the remains of tracer dye after three weeks of intensive treatment in 47.4% of the cases under study. We found that this result was associated with a single variable: lymphedema evolution time. Lymphedemas that were more than one year old were more likely (80%) to demonstrate ICG tracer remains despite three weeks of intensive treatment. After three weeks of intensive physiotherapy treatment, the patterns that improved did so as follows: the diffuse pattern (severe) evolved in 60% of cases (14 areas) to a splash pattern (mild) and, in 36% of cases (9 areas), to a linear pattern. The stardust pattern (moderate) evolved in 70% of cases (15 areas) toward a slight pattern or splashes and the rest (6 areas) toward a linear pattern. ICG patterns that were slight (splash) remained unchanged and stable in 43% of cases (12 areas) after intensive therapy. These data can be consulted in in the Table S4 of the supplementary material of this manuscript.

The worsening of ICG patterns after physical therapy treatment in our study was rare (7%). It happened in three cases and was observed in the forearm 73% of the time. 

## Figures and Tables

**Table 1:** Relationship in absolute frequencies, between the time of duration of the lymphedema and the visibility of the indocyanine green (ICG) tracer.

Duration of Lymphedema	Group 1 (No Tracer Remains After Treatment) *N* = 10	Group 2 (Present Tracer Remains After Treatment) *N* = 9	Total	*p*-Value
<3 months	3	0	3	≤0.001
3–6 months	5	1	6	
>1 year	2	8	10	
Total	10	9	19	

**Table 2:** Relationship in absolute frequencies and percentages, between the level of initial severity of the lymphedema and the visibility of the ICG tracer.

Level of Initial Severity of Lymphedema	Group 1 (No Tracer Remains After Treatment) *N* = 10	Group 2 (Present Tracer Remains After Treatment) *N* = 9	Total	*p*-Value
Mild	6 100%	0 0%	6 31.6%	≤0.021
*Moderate*	2 28.6%	5 71.4%	7 36.8%	
Most severe	2 33.3%	4 66.7%	6 31.6%	
Total	10	9	19	

## Supplementary Materials

The following are available online at https://www.mdpi.com/2077-0383/9/2/306/s1. It include: the evolution of the ICG pattern in the anterior and posterior regions of the hand and wrist (Table S1), forearm (Table S2), and from the elbow to the armpit (Table S3), at baseline and after the physiotherapy treatment, and the results, in absolute frequencies, of the changes of ICG pattern in the body regions assessed after the physiotherapy treatment (Table S4).
