# Supplementary material for: Changes in Indocyanine Green Lymphography Patterns after Physical Treatment in Secondary Upper Limb Lymphedema
Source: J Clin Med. 2020 Jan 22;9(2):306. doi: 10.3390/jcm9020306 (PMC7073737; doi:10.3390/jcm9020306)
Supplement: Supplementary file 1 [file jcm-09-00306-s001.pdf]

Supplementary Table 1. Evolution of the ICG pattern in the anterior (ANT) and posterior (POST) regions of the hand and wrist at baseline (B) and after the Physiotherapy (A) treatment, in the 10 subjects under study.

ID	*PALM OF HAND*			*BACK OF HAND*			*ANTERIOR WRIST*			*POSTERIOR WIRST*		
	B	*A*	*EV[1]*	*B*	*A*	*EV[1]*	*B*	*A*	*EV[1]*	*B*	*A*	*EV[1]*
1	0	0	*RU*	1	1	*RU*	0	0	RU	1	1	RU
2	0	0	*RU*	1	1	RU	0	0	RU	1	1	RU
3	0	1	*NVa*	1	1	RU	0	1	*NVa*	1	1	RU
4	0	0	*RU*	1	1	RU	1	0	*NVa*	1	1	RU
5	1	1	*RU*	4	1	*I*	4	1	*I*	4	2	*I*
6	0	0	*RU*	1	1	RU	4	1	*I*	1	1	*RU*
7	4	3	*I*	4	4	RU	3	3	RU	4	2	*I*
8	0	0	*RU*	4	1	*I*	4	4	RU	4	2	*I*
9	0	0	*RU*	1	1	*RU*	4	1	*I*	1	1	*RU*
10	4	0	*NVa*	3	1	*I*	4	2	*I*	2	3	*W*

(0) Not observed, (1) linear pattern, (2) splash pattern, (3) stardust pattern , (4) diffuse pattern; EV1: Evolution of the pattern.. RU: remained unchanged., NVa: Not valuable, I: improved, P: worsened. .

Supplementary Table 2. Evolution of the ICG pattern in the anterior (ANT) and posterior (POST) regions of the forearm (at 4, 8, 12 and 16 cm of the wrist) at baseline (B) and after Physiotherapy treatment (A) in the ten subjects studied.

BD	***To 4 cm of the wrist. Ant***			***To 4 cm of the wrist... Post.***			***To 8 cm of the wrist. Ant.***			***To 8 cm of the wrist. Post.***			***To 12 cm of the wrist. Ant.***			***To 12 cm of the wrist... Post.***			***To 16 cm of the wrist. Ant.***			***To 16 cm of the wrist. Post.***		
	B	*A*	*EV[1]*	*B*	*A*	*EV[1]*	*B*	*A*	*EV[1]*	*B*	*A*	*EV[1]*	*B*	*A*	*EV[1]*	*B*	*A*	*EV[1]*	*B*	*A*	*EV[1]*	*B*	*A*	*EV[1]*
1	0	0	*RU*	2	0	*NVa*	4	0	*NVa*	4	0	*NVa*	2	0	*NVa*	4	0	*NVa*	2	0	*NVa*	2	0	*NVa*
2	0	1	*NVa*	1	1	*RU*	0	0	*RU*	1	0	*NVa*	0	0	*RU*	1	0	*NVa*	4	0	*NVa*	4	0	*NVa*
3	0	1	*NVa*	4	1	*I*	4	1	*I*	1	1	*RU*	4	1	*I*	1	1	*RU*	2	1	*I*	1	1	*RU*
4	4	4	*RU*	3	1	*I*	2	4	*W*	3	3	*RU*	2	4	*W*	2	1	*I*	2	3	*W*	2	1	*I*
5	3	4	*W*	4	2	*I*	3	4	*W*	3	2	*I*	3	2	*I*	3	4	*W*	3	4	*W*	3	4	*W*
6	0	1	*NVa*	1	1	*RU*	3	3	*RU*	3	1	*I*	3	3	*RU*	3	1	*M*	0	2	*NVa*	3	1	*I*
7	3	3	*RU*	4	2	*I*	3	3	*RU*	4	2	*I*	3	2	*M*	2	2	*RU*	3	2	*MI*	2	0	*NVa*
8	4	4	*RU*	2	2	*RU*	2	4	*W*	2	2	*RU*	2	2	*RU*	2	2	*RU*	1	2	*W*	1	2	*W*
9	2	2	*RU*	1	1	*RU*	2	2	*RU*	2	2	*RU*	4	2	*I*	1	1	*RU*	1	1	*RU*	1	1	*RU*
10	3	2	*I*	4	4	*RU*	1	0	*NVa*	2	0	*NVa*	0	1	*NVa*	0	2	*NVa*	2	1	*I*	0	0	*RU*

(0) Not observed pattern, (1) linear pattern, (2) splash pattern, (3) stardust pattern , (4) diffuse pattern; EV[1]: Evolution of the pattern.. RU: remained unchanged., NVa: Not valuable, I: improved, P: worsened.

Supplementary Table 3. Evolution of the ICG pattern in the anterior (ANT) and posterior (POST) regions of the elbow to the amrpit (at 4, 8,12 and 16 cm. over the elbow) at baseline (B) and after the Physiotherapy treatment (A) in the ten subjects studied.

ID	*Elbow Ant.*			*Elbow Post.*			*To 4 cm over elbow Ant*			*To 4 cm over elbow Post.*			*To 8 cm over elbow Ant.*			*To 8 over elbow Post.*			*To 12 cm over elbow Ant.*			*To 12 cm over elbow Post.*			*To 16 cm over elbow Ant.*			*To 16 cm over elbow Post.*		
	B	*A*	*EV[1]*	*B*	*A*	*EV[1]*	*B*	*A*	*EV[1]*	*B*	*A*	*EV[1]*	*B*	*A*	*EV[1]*	*B*	*A*	*EV[1]*	*B*	*A*	*EV[1]*	*B*	*A*	*EV[1]*	*B*	*A*	*EV[1]*	*B*	*A*	*EV[1]*
1	0	0	RU	2	1	I	3	2	M	2	2	RU	3	2	I	2	2	RU	3	2	M	4	2	I	4	0	NVa	4	2	I
2	2	2	RU	4	4	RU	2	2	RU	2	2	RU	2	2	RU	2	2	RU	2	2	RU	2	0	NVa	2	2	RU	2	0	NVa
3	1	1	RU	4	2	I	1	1	RU	1	1	RU	2	1	I	1	1	RU	1	1	RU	2	1	I	1	1	RU	2	1	I
4	2	1	I	2	1	I	2	2	RU	3	1	I	3	2	I	3	2	I	3	0	NVa	3	2	I	3	0	NVa	2	2	RU
5	0	4	NVa	3	3	RU	2	4	W	3	3	RU	2	4	W	3	4	W	2	0	NVa	4	0	NVa	0	0	RU	0	0	RU
6	1	1	RU	0	0	RU	3	2	M	0	2	NVa	3	2	I	0	0	RU	3	3	RU	0	0	RU	3	3	RU	0	0	RU
7	1	0	NVa	2	1	I	1	0	NVa	0	4	NVa	1	0	NVa	0	0	RU	1	0	NVa	0	0	RU	1	0	NVa	0	0	RU
8	4	0	NVa	4	2	I	0	0	RU	3	2	I	4	0	NVa	4	2	I	4	0	NVa	4	2	I	4	0	NVa	0	0	RU
9	1	1	RU	2	1	I	1	0	NVa	4	1	I	1	0	NVa	0	0	RU	0	0	RU	0	0	RU	0	0	RU	0	0	RU
10	2	2	RU	4	4	RU	2	2	RU	2	2	RU	0	0	RU	0	2	NVa	0	0	RU	4	2	I	0	0	RU	4	4	RU

(0) Not observed, (1) linear pattern, (2) splash pattern, (3) stardust pattern , (4) diffuse pattern; EV1: Evolution of the pattern.. RU: remained unchanged., NVa: Not valuable, I: improved, P: worsened.

Supplementary Tabla 4. Results in absolute frequencies of the changes of ICG pattern in the body regions assessed, after the Physiotherapy treatment.

		ABSOLUTES FRECUENCIES OF THE CHANGES IN THE PATTERNS									
		**IMPROVED**						**WORSENED**			
BODY REGION		**4 to 3**	**4 to 2**	**4 to 1**	**3 to 2**	**3 to 1**	**2 to 1**	**3 to 4**	**2 to 4**	**2 to 3**	**1 to 2**
HAND	***PALM***	**1**									
	BACK			**2**		**1**					
WRIST	***ANT***		*1*	*3*							
	POST		*3*							*1*	
FOERARM — ***To 4 cm. over the wrist***	***ANT.***				*1*			*1*			
	POST.		*2*	*1*		*1*					
FOERARM — ***To 8 cm. over the wrist.***	***ANT.***			*1*				*1*	*2*		
	POST.		*1*		*1*	*1*					
FOERARM — ***To 12 cm. over the wrist .***	***ANT.***		*1*	*1*	*2*				*1*		
	POST.					*1*	*1*	*1*			
FOERARM — ***To 16 cm. over the wrist.***	***ANT.***				*1*		*2*	*1*		*1*	*1*
	POST.					*1*	*1*	*1*			*1*
ELBOW	***ANT.***						*1*				
	POST.		*2*				*4*				
ARM — ***To 4 cm. over elbow.***	***ANT.***				*2*				*1*		
	POST.			*1*	*1*	*1*					
ARM — ***To 8 cm. over elbow.***	***ANT.***				*3*		*1*		*1*		
	POST.		*1*		*1*			*1*			
ARM — ***To 12 cm. over elbow.***	***ANT.***				*1*						
	POST.		*3*		*1*		*1*				
ARM — ***To 16 cm. over elbow.***	***ANT.***										
	POST.				*1*		*1*				
TOTAL (ABSOLUTE FREQUENCIES)		*1*	*14*	*9*	*15*	*6*	*12*	*6*	*5*	*2*	*2*

(1) linear pattern, (2) splash pattern, (3) stardust pattern, (4) diffuse pattern.
